# Supplementary material for: A Global Screen for Assembly State Changes of the Mitotic Proteome by SEC-SWATH-MS
Source: Cell Syst. 2020 Feb 26;10(2):133–155.e6. doi: 10.1016/j.cels.2020.01.001 (PMC7042714; doi:10.1016/j.cels.2020.01.001)

Q9UDX5 | MTFP1_HUMAN | MTFP1 MTP18 HSPC242 My022
Monomer MW [kDa]: 18.01 Monomer expected elution fraction: 55

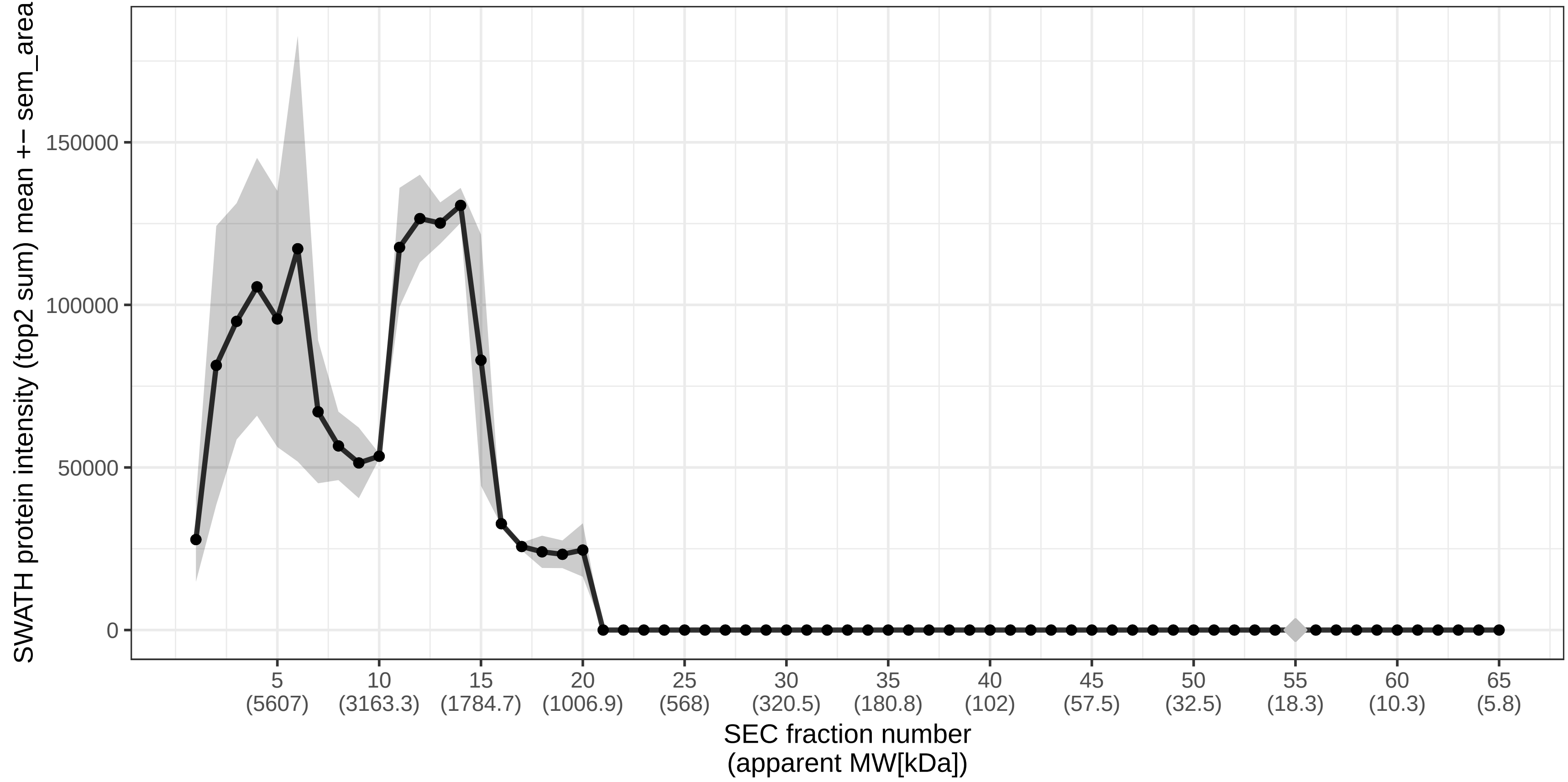

Supplement: Data S1. SEC-SWATH-MS Protein Chromatograms, Related to Figure 1 [file mmc6.zip › SECchrom_Q9UDX5_MTFP1_HUMAN_MTFP1_MTP18_HSPC242_My022.pdf]
